# Molecular characterization of fire ants, *Solenopsis* spp., from Brazil based on analysis of mtDNA gene cytochrome oxidase I

**DOI:** 10.1093/jis/14.1.50

**Published:** 2014-01-01

**Authors:** Cintia Martins, Rodrigo Fernando de Souza, Odair Correa Bueno

**Affiliations:** 1 Universidade Federal do Piauí, Campus de Parnaíba, Avenida São Sebastião, 2819, 64202-020, Parnaíba, Piauí, Brazil; 2 UNESP Universidade Estadual Paulista Julio de Mesquista Filho/ CEIS Centro de Estudos de Insetos Sociais. Avenida 24A, 1515. CEP 13506-900 Rio Claro, São Paulo, Brazil; 3 UNESP Universidade Estadual Paulista Julio de Mesquista Filho/ CEIS Centro de Estudos de Insetos Sociais. Avenida 24A, 1515. CEP 13506-900 Rio Claro, São Paulo, Brazil

**Keywords:** mitochondrial DNA, phylogeny, *Solenopsis invicta*, *Solenopsis saevissima*

## Abstract

Species from the *Solenopsis saevissima* (Smith) (Hymenoptera: Formicidae) species group are native to South America and have a cosmopolitan distribution because they have been accidentally introduced in many countries around the world. In Brazil, they have a wide distribution, including urban areas. The present study was conducted to investigate the characterization of *Solenopsis* genus populations associated with urban/human interference sites in Brazil by analyzing the mitochondrial gene cytochrome oxidase I and estimating the degree of relatedness of these populations to make inferences about their phylogeny and also observe the patterns of mitochondrial haplotype (mitotype) distribution across their range. The results revealed complete geographical coherence and polyphyly for the *Solenopsis invicta* Buren and *Solenopsis saevissima* species groups, which confirms the diversity of the genera. It also suggests the possibility that reproductively-isolated populations occur, resulting in the evolutionary process of speciation. No predominant haplotype was found in the populations analyzed, but some were more prevalent.

## Introduction

Ants are highly adaptive insects and are distributed in most terrestrial environments in great abundance and diversity. Many species have aggressive behavior, which in turn may displace other species. Invasive species can handle several types of habitats, such as urban and agricultural areas, that are of great social and economic importance to humans. In this invasive group are the ants of the genus *Solenopsis* (including the well-known fire ant), which occur worldwide and have a wide distribution in Brazil, including in urban areas. They are highly aggressive in defense of their colony and during foraging.

The species *Solenopsis invicta* Buren (Hymenoptera: Formicidae) was spread from South America to various places around the world via wood export ( Taber 2000 ). Their presence has been documented in the United States, the West Indies, New Zealand, Puerto Rico, Australia ( Henschaw et al. 2005 ; Tschinkel 2006 ), Taiwan ( Chen et al. 2006 ), and China ( Yijuan et al. 2007 ).

In South America, a place of high ant genera diversity, the distinction between *Solenopsis* species is difficult due to a reduced number of diagnostic characteristics ( Pitts et al. 2005 ). An important study by Pitts et al. (2005) about cladistic analysis of the *Solenopsis saevissima* (Smith) species-group represented a step towards understanding the group, but some important unresolved issues remained (see Shoemaker et al. 2006 ).

Wilson (1952) considered that there may be only three species of fire ants, with South American fire ants comprising a large hybrid ant colony with several variants of hybridizations of parapatric regional areas.

Ross and Shoemaker (2005) conducted a genetic analysis to delineate species of fire ants in South America and found that *S. invicta* and *S. richteri* were reproductively isolated, in contrast to previous findings suggesting the existence of regions in which hybrid colonies existed in the USA ( Shoemaker et al. 1996 ). In addition, it was proposed that the existence of cryptic species in *S. invicta* and *S. richteri* indicated that the group was undergoing radiation and morphological differences that were not leading to reproductive isolation or neutral genetic divergence. It is noteworthy that before Ross and Shoemaker (2005) , the occurrence of cryptic species of *S. invicta* were found by observed divergences in mitochondrial DNA by Shoemaker et al. 2003b , and more recently Delsinne et al. (2012) also suggested the existence of cryptic species in *Solenopsis* genus inferred by cytochrome oxidase I (COI) and nuclear wingless genetic markers.

Phylogenetic analysis carried out by Shoemaker et al. (2006) for the species group *Solenopsis saevissima* based on mitochondrial DNA sequences of samples from Brazil and Argentina imply that the group should be monophyletic. However, they found an occurrence of divergent mitochondrial DNA lineages in several species, suggesting a polyphyletic pattern for the invasive *S. invicta.*

According to Ross et al. (2009) , high levels of evolutionary divergence and differentiation between regional populations of *S. saevissima* do occur. As these two widely distributed populations are connected by substantial levels of recent gene flow, other groups are evolutionarily independent or on the way to becoming such. Several of these lineages are parapatric with other populations, suggesting that intrinsic barriers to premating and post-mating gene flow are occurring. Ross et al. (2009) also suggested that genetic differences found in *S. saevissima* might be due to interspecific hybridization with other regional species that occur in sympatry or parapatry, including *S. geminata* .

Considering that South America is the focus of fire ant occurrence, two aspects are relevant: 1) the Pantanal region of South America is considered the nucleus of dispersion of *S. invicta* , and 2) the other regions of Brazil are dominated by *S. saevissima* ( Ahrens et al. 2005 ; Ross and Shoemaker 2005 ; Shoemaker et al. 2006 ; Ross et al. 2009 ).

Despite the wide distribution of fire ants throughout the world and several studies focusing on understanding their evolution and distribution aspects, there are no specific studies of their distribution in urban or human interfered habitats in Brazil, which is part of their place of origin.

The aims of this study were to characterize the populations of fire ants ( *Solenopsis* spp.) from several regions of Brazil and Corrientes, Argentina, through analysis of mitochondrial DNA gene sequences, including part of the COI gene. The focus populations from this analysis were fire ants associated with urban or human-interfered habitats. Through phylogenetic analysis, the degree of relatedness of these populations was determined and their phylogeny was inferred. We first expected to find a prevalent haplotype associated with urban habitats. However, geographical coherence was found in *S. invicta* and *S. saevissima* , but no predominant haplotype was found in the populations analyzed, which clearly illustrates the diversity of the genera in Brazil.

## Materials and Methods

### Specimen collection, identification, and material preservation

**Figure 1.**
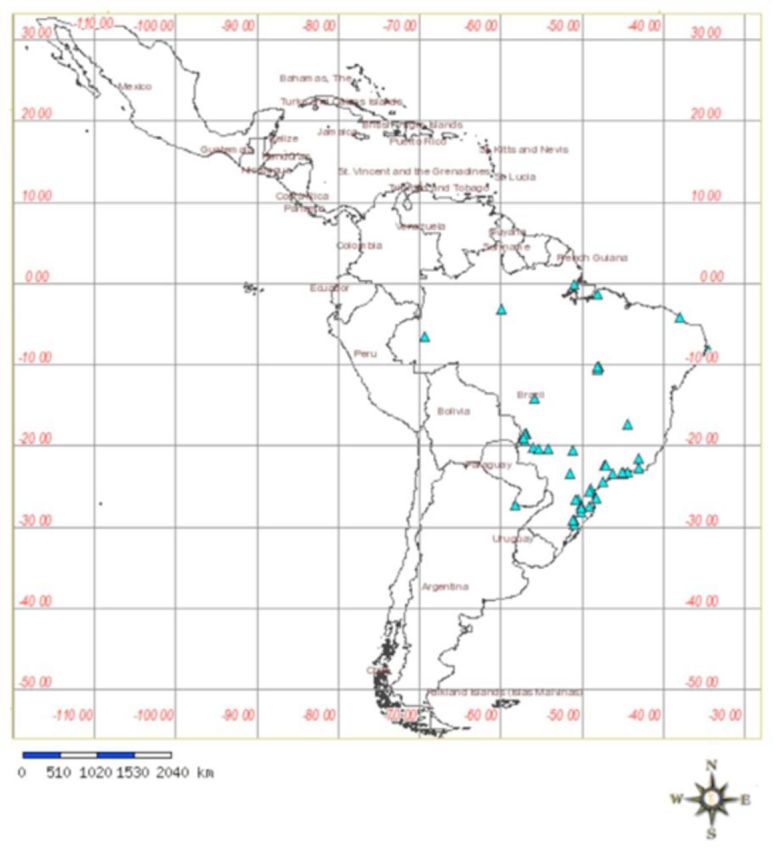
South America map showing the 42 localities of collection of *Solenopsis* spp. Table 1 shows details. Map created with speciesMapper ( http://splink.cria.org.br/mapper?criaLANG=pt ). High quality figures are available online.

The 114 analyzed sample nests were collected by the authors at 42 locations in Brazil, in the states of Amapa, Amazonas, Para, Tocantins, Mato Grosso do Sul, Minas Gerais, Parana, Rio Grande do Sul, Santa Catarina, and Sao Paulo, as well as samples from Corrientes, Argentina. The collected samples were associated with habitats that had been interfered with or disturbed by humans. Table 1 includes the collection codes, locations, species, mitochondrial DNA haplotype, geographic coordinates, and correspondent GenBank accession numbers. The collection sites are shown in Figure 1 .

**Table 1.**
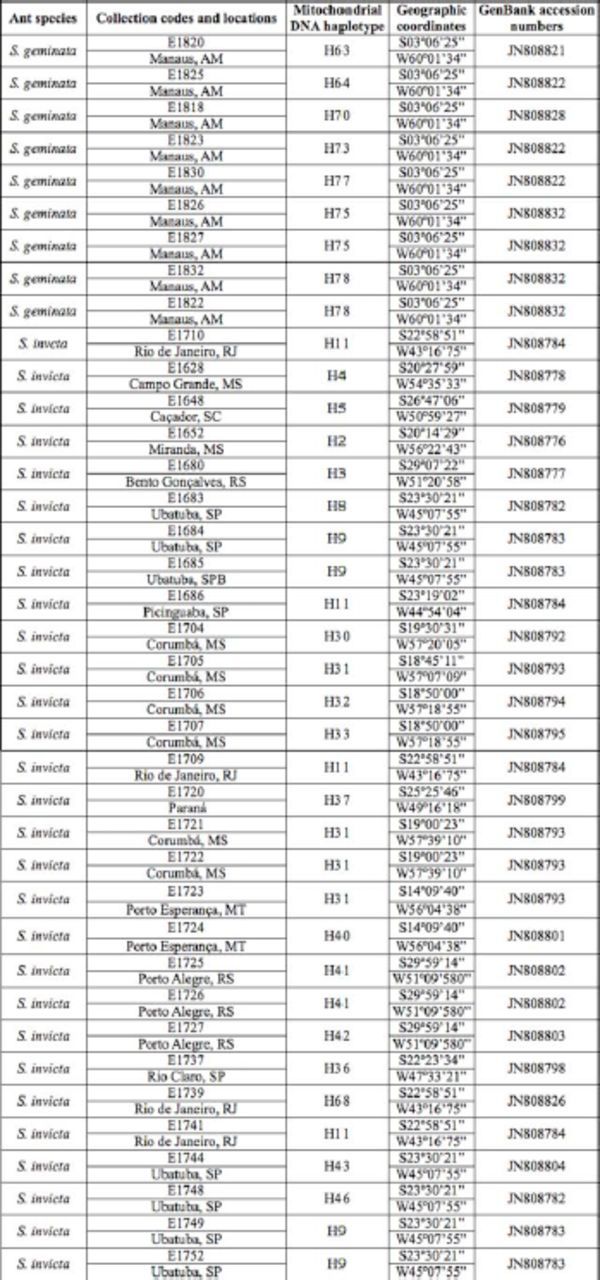

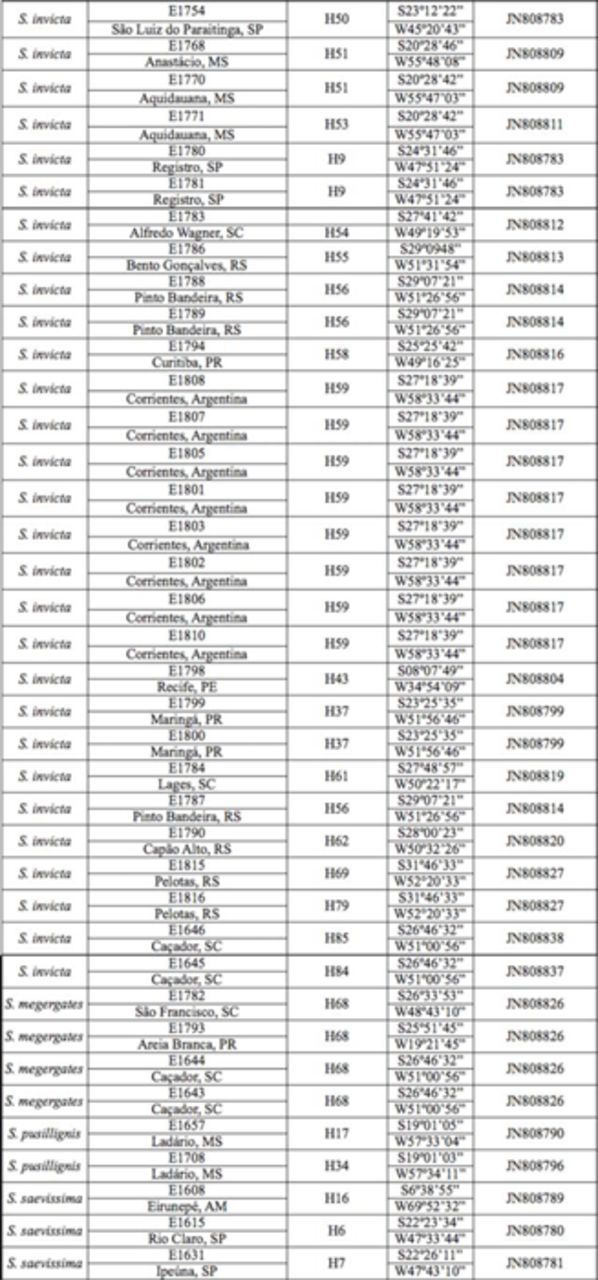

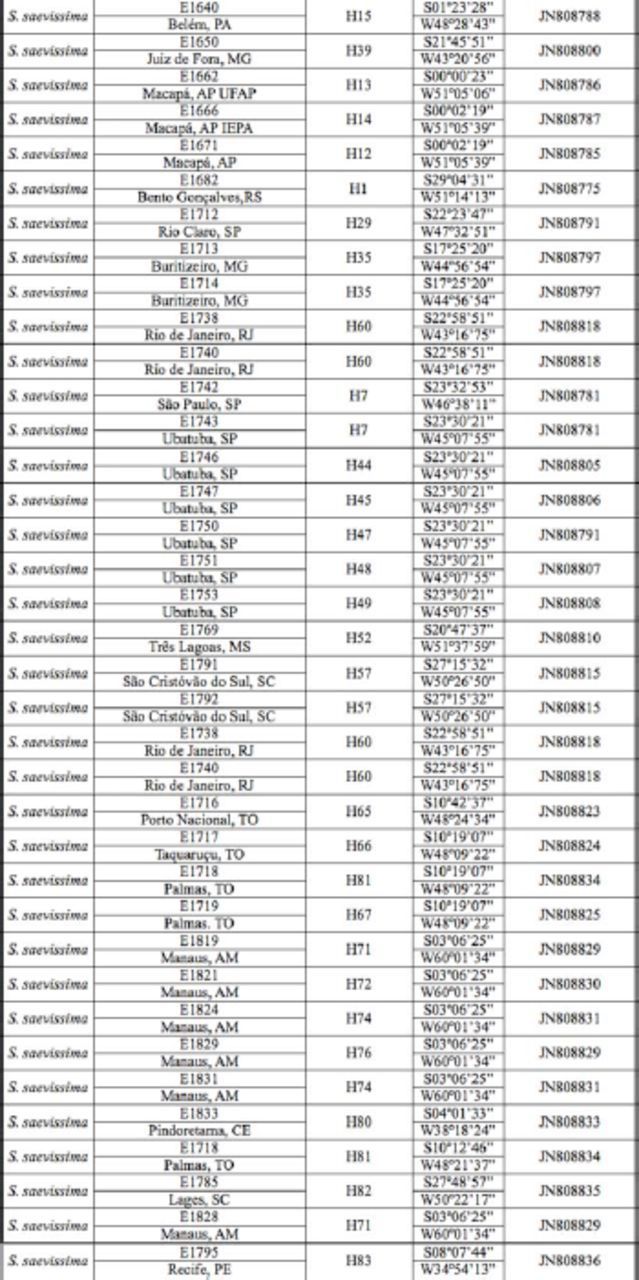
Ant species, collection codes and locations, mitochondrial DNA haplotype, geographic coordinates, GenBank accession numbers.

The samples contained workers of various sizes that were fixed and maintained in 80% ethanol under freezing to prevent degradation of DNA until the moment of use. The identification was made based on Trager (1991) and Pitts (2002) .

The visual differentiation between different species of *Solenopsis* is hampered due to poor definition of morphological characteristics ( Pitts et al. 2005 ). In this sense, molecular data can clarify the doubts created by morphological identifications and may even be the main tool used to differentiate species by allowing for the creation of a DNA barcode ( Hebert et al. 2003a ; Hebert et al. 2003b ; Ratnasingham and Hebert 2007 ; Hebert et al. 2010 ). According to Pitts (2002) , there seems to be higher-level concordance between mtDNA data and the morphological data in some *Solenopsis* species. In this sense, we used mitochondrial DNA data, more specifically COI, for species identification confirmation. By sequencing part of the COI gene, fragments were generated for all populations. Then, using the NCBI Blast (National Center for Biotechnology Information, www.ncbi.nlm.nih.gov ), we compared our data with sequences deposited in GenBank. Species identity was confirmed when there was great similarity of the experimental and database sequences; this was defined as either high score values or Evalues equal to or close to 0 or very close to those deposited in the database.

### DNA extraction

Total DNA was extracted using a non-phenolic method. Five whole ant workers (pooled) were used. The extraction protocol was the same as used in Martins et al. (2010) .

### PCR amplification

Mitochondrial DNA fragments of approximately 920 bp were amplified by PCR. These fragments were part of the COI gene (approximately 780 bp), leucine transfer RNA (70 bp), and part of the cytochrome oxidase II (approximately 60 bp). The amplifications were carried out with a final volume of 25 µL, containing 250 to 500 ng of DNA template and 0.2–0.4 µM (5–10 pmol) of each primer, using the Ready-to-go kit (Amersham Pharmacia Biotech, GE Healthcare Life Sciences, www.gelifesciences.com ).

The thermal cycler was programmed as proposed by Ross and Shoemaker (1997) : 1 min at 94ºC (initial denaturation) and 35 cycles at 94ºC for 1 min, annealing temperature of 48ºC for 1 min, and extension temperature of 68ºC for 2 min, followed by a final extension step at 72ºC for 5 min.

The primers used were: C1-J-2195 (COI-RLR) (5’ – TTGATTTTTTGGTCATCCAG AAGT -3’) and DDS-COII-4 (5’ – TAAGAT GGTTAATGAAGAGTAG -3’) ( Ahrens et al. 2005 ; Ross and Shoemaker 1997 ). When the combination of primers did not amplify the desired fragment, a second primer was used instead of DDS-COII-4, named Jer-ryGarcia-CI (5’ – GGGAATTAGAATTTTG AAGAG – 3’) ( Shoemaker et al. 2006 ), which produces fragments of approximately 780 bp that include only the gene COI.

### DNA sequencing

DNA was sequenced with the BigDye Terminator Kit (Applied Biosystems, Life Technologies, www.lifetechnologies.com ). Both DNA chains of each sample were sequenced separately with the corresponding primers using an automatic sequencer ABI Prism 377 (Applied Biosystem). DNA sequencing was carried out according to standard protocols. The final volume was 10 µL. The extension products were precipitated with 75% isopropanol.

### Phylogenetic analysis

The sequences were initially analyzed separately with BioEdit software ( www.mbio.ncsu.edu/BioEdit/bioedit.html ) and aligned using ClustalW software ( Higgins et al. 1992 ) followed by manual modifications. A second and more refined alignment was performed with MUSCLE3.6 software ( Edgar 2004 ).

After all sequences were aligned with the sequences retrieved from GenBank (Table 4), some bases at the end of the fragment were excluded due to unsatisfactory alignment. The resulting matrix consisted of approximately 700 bp comprising only the COI.

The resulting alignment was used for the construction of the network of strains using DnaSP4.90 software ( Rozas et al. 2003 ) and Network4.5 ( www.fluxus-engineering.com ) using the median joining parameter ( Bandelt et al. 1999 ).

The reconstruction of the phylogeny based on maximum parsimony analysis was conducted using PAUP 4.0 software ( Swofford 2003 ). The data set was analyzed using setting 1 for gap and setting 3 for substitutions. One thousand replicates were used to generate bootstrap values.

MrModeltest 2.2 ( Nylander 2002 ) was used before carrying out Bayesian analyses, appropriate models of sequence evolution were chosen via the Akaike information criterion, and the model selected was GTR+I+G. The reconstruction of the phylogeny based on the Bayesian analysis was carried out using MrBayes software ( Huelsenbeck et al. 2001 ). A Markov chain was run for 1,000,000 generations and sampled at each 100 generations. To summarize the parametric values and the trees generated, the first 10% of the trees were excluded as burnin, and the probability values were then calculated with the remaining trees.

Considering the clade division found in phylogenetics analysis, we analyzed clade 3, clade 5, clade 6, and clade 7 in terms of haplotype diversity, nucleotide diversity, Tajima’s D, polimorphic sites, G+C content, and average number of nucleotide differences between populations with DnaSP4.90 software ( Rozas et al. 2003 ).

## Results

**Figure 2.**
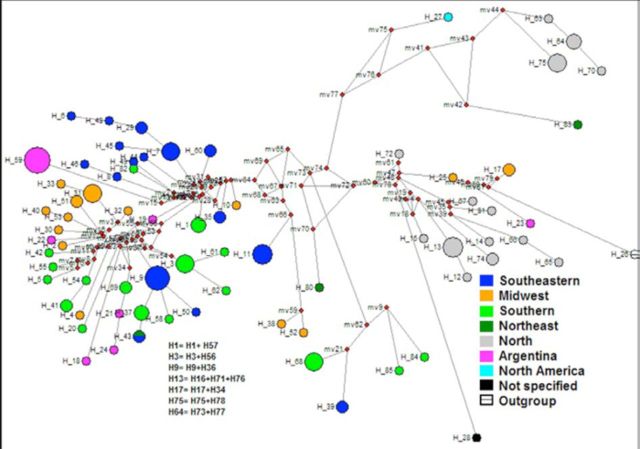
Network of haplotypes of mtDNA from the 114 *Solenopsis* populations studied. The red dots indicate hypothetical ancestors. High quality figures are available online.

Of the 114 analyzed colonies, 72 had a unique haplotype sequence of the COI mitochondrial DNA, which are illustrated in the network ( Figure 2 ). Table 1 includes the species identification, the collecting locales (geo-referenced), and the corresponding haplotypes. All COI sequences generated in this study have been deposited in the GenBank database under accession numbers JN808775 to JN808838 (see Table 1 ).

The prevalent haplotypes were H59 (Argentina); H7, H9, and H11 (in southeastern Brazil); H31 (midwest Brazil); H68 and H3 (southern Brazil); and H13, H64, and H75 (northern Brazil). Furthermore, the remaining haplotypes did not seem to have derived from the most prevalent ones (see Figure 2 ). Moreover, the haplotype distribution in the network indicates that there was no shared haplotypes between different localities, suggesting great diversity of *Solenopsis* in Brazil, but now seen in a view from those ants associated with human-disturbed habitats.

**Figure 3.**
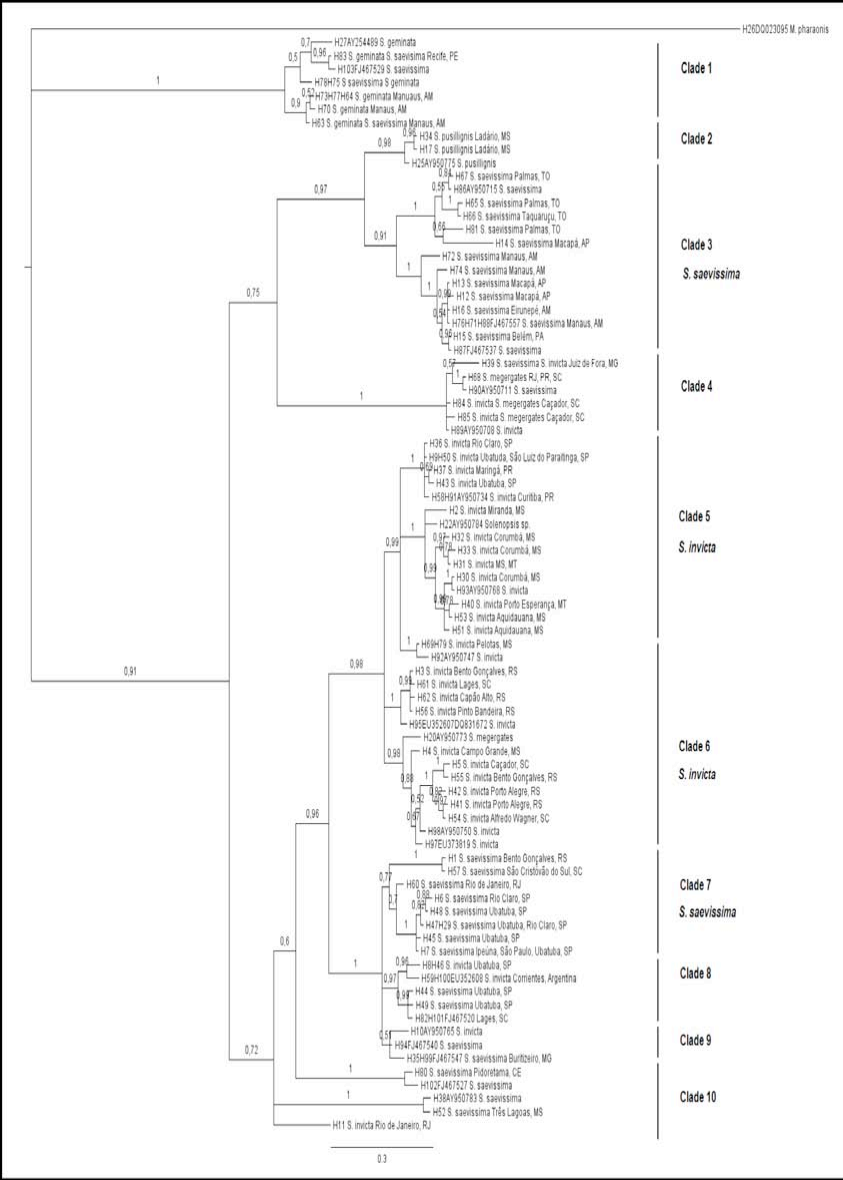
Bayesian inference tree reconstructed from the COI gene in populations of *Solenopsis* from different regions of Brazil and part of Argentina. Posterior probability values are shown above branches. The tree was rooted with a representative of *Monomorium pharaonis* . High quality figures are available online.

Of the 726 characters used in maximum parsimony analysis, 482 were constant and 206 were informative characters (parsimony-informative). Forty-eight equally parsimonious trees were found as a result of phylogenetic analysis of different *Solenopsis* populations based on a portion of the COI gene. Both analyses (maximum parsimony and Bayesian inference) were nearly the same, and only the Bayesian tree analysis was illustrated with posterior probability values ( Figure 3 ).

The phylogenetic tree was rooted with a representative of the species *Monomorium pharaonis* recovered from GenBank. Several internal sequences from GenBank of some species of *Solenopsis* were also incorporated into the analysis ( Table 2 ). The clusters in the phylogenetic tree ( Figure 3 ) revealed the occurrence of several clades, and most are well supported. Important clades include the following:

**Table 2.**
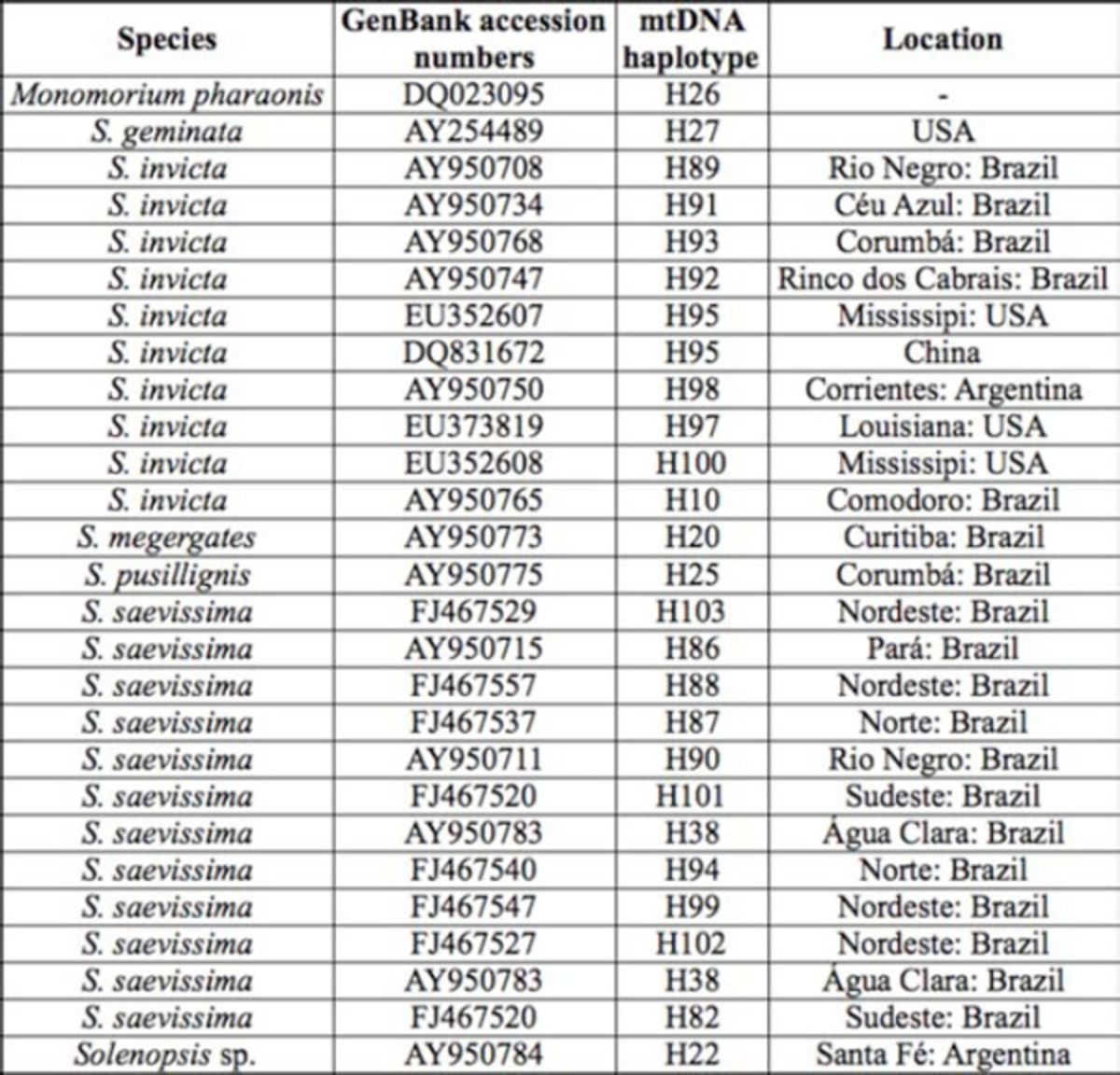
Ant species used as outgroup and in-group in phylogenetic analyses and respective GenBank accession numbers, designed haplotypes from these analyses, and collection location retrieved from data in GenBank.

Clade 1: The presence of diverging species grouped as closely-related species disagrees with the phylogeny proposed by Shoemaker et al. (2006) and Tschinkel et al. (2006) . The two species *S. geminata* and *S. saevissima* are morphologically distinct, even though they have been grouped in this clade.

Clade 2: Representatives of *S. pusilligni* , collected in Ladário, Mato Grosso do Sul, cluster with the representative of the same species recovered from GenBank (AY950775).

Clade 3: Representatives of *S. saevissima* populations from the northern region of Brazil along with representatives listed in GenBank (AY950715, FJ467557, FJ467537). This clade appears to differ from populations of *S. saevissima* from southern Brazil (clade 7).

Clade 4: Once again, the presence of diverging species grouped as closely-related species ( *S. saevissima* , *S. megergates* , and *S. invicta* ).

Clade 5: Representatives of *S. invicta* populations from the states of São Paulo, Paraná, Mato Grosso, and Mato Grosso do Sul form a well-supported clade of specimens of *S. invicta* with restricted occurrence to this geographical area.

Clade 6: A second clade of *S. invicta* occurs in populations from the state of Rio Grande do Sul and Santa Catarina.

Clades 8, 9, and 10: Representatives of the *S. invicta* and *S. saevissima* species form an isolated group of representatives of these species that are not allocated in previous clades. The terminal clade contains the representative of *S. invicta* from the Rio de Janeiro.

The results of this analysis reveal the existence of well-supported clades of *S. invicta* and *S. saevissima* from different geographical regions that are split between *S. saevissima* that occur in the North, South, southern, and Midwest Brazil. *S. invicta* also has separate representatives from the South and Southeast.

G+C content of the samples was approximately 30%, corroborating the high A+T frequencies expected for insects ( Simon et al. 1994 ). The average number of nucleotide differences between clades 5 and 6 was 18,420 and between clades 3 and 7 was 65,229.

The number of haplotypes found in clade 7 was the lowest of all. As for haplotype diversity, clade 3 was the lowest, followed by clade 5, 7, and 6. Regarding nucleotide diversity (π), clade 7 was the lowest, followed by 5, 6, and 3. Tajima’s D for clade 3 and 6 was negative (-0.02978 and -0.18128 respectively) and for clade 5 and 7 was positive (0.13862 and 0.36991 respectively). Clade 7 was the one with the lowest polymorphic sites, followed by clade 5, 6, and 3 (see Table 3 to summarize results).

**Table 3.**
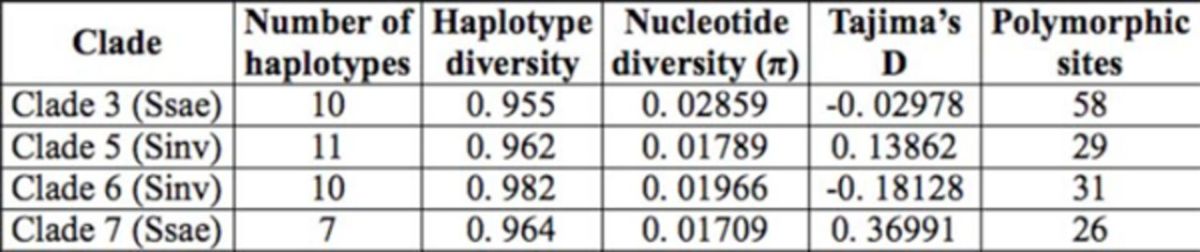
Number of haplotypes for each clade, haplotype diversity, nucleotide diversity, Tajima’s D, and polymorphic sites.

Ssae = *S. saevissima* , Sinv = *S. invicta* .

## Discussion

The results show complete consistency when grouping populations according to geographical distribution and even polyphyly for *S. invicta* and *S. saevisisma,* which reveals diversity of this ant genus in Brazil. However grouping of divergent species (see clades 1, 4, 6, 8, and 9 in Figure 3 ) could be due to a lack of data collection in a region where representatives of these species occur, which could bring together representatives to form new clades, such as those found by Shoemaker et al. (2006) , or indicative that those haplotypes that could form clades not supported here rarely occur in urban areas.

The polyphyly found in *S. invicta* ( Figure 3 ) was also observed by Shoemaker et al. 2000 , 2003, and 2006. Shoemaker et al. (2006) found discordance between the phylogeny reconstructed with mtDNA haplotypes and those constructed using morphology, and they reported seven well-supported clades of the species *S. invicta* . They suggested that the polyphyly of the mitochondrial DNA sequence of these species appears to result in the crowding of multiple morphological characteristics that represent genetic lineages that are evolutionarily indistinguishable and independent (cryptic species). They concluded that current morphological boundaries overestimate the distribution of fire ants and assumed that the mtDNA tree they reconstructed faithfully categorized the development of reproductive isolation and patterns of ancestral populations of the *S. saevissima* species group.

The geographical grouping of *S. invicta* (clades 5 and 6) and *S. saevissima* (clades 3 and 7) supports the hypothesis that regional populations of each species are derived from refuges or large isolated areas of earlier endemism from which expansion has occurred ( Ahrens et al. 2005 ; Shoemaker et al. 2006 ). Because our focus was populations from *Solenopsis* from habitats that were affected by humans, this expansion could be driven by human activities.

Climate patterns could also be the cause of the presence of divergent clades of *S. saevissima* (clades 3 and 7). The third clade has representatives from the states of Tocantins, Amazonas, Pará, and Amapá, which are characterized by hot climates from semi-humid (Tocantins) to wet (Amazonas, Pará, and Amapá) located within the Amazon biome; the seventh clade has representatives from the states in southern Brazil where the climate is predominantly hot (São Paulo) and mid-mesothermal (Rio Grande do Sul and Santa Catarina). On the other hand, the high nucleotide diversity observed in clade 3 (D < 0) could be related to geographic distribution because the northern region of Brazil is less populated, so human interference is reduced, which in turn can reduce the pressure against divergence of those ants populations consequently experiencing rapid growth.

The presence of the endosymbiont *Wolbachia* can influence cytoplasmic genome selection, such as on host mtDNA evolution ( Shoemaker et al. 2003 ). The presence of *Wolbachia* may be related to the divergence of the *S. invicta* and *S. saevissima* clades in the present study. It may indicate traces of increased substitution rates in mtDNA associated with recurrent *Wolbachia* infections in affected lineages ( Shoemaker et al. 2004 ). For this scenario to be possible, separate clades of concerned species should be fully isolated or have minimal evidence of migration, which should be the case for the populations studied herein that are geographically separated by many miles. Additionally, *Solenopsis* populations from Brazil with high rates of *Wolbachia* infection offset other populations in which the infection rate is low or absent ( Martins et al. 2012 ).

Populations from *S. saevissima* comprising clade 3 show D < 0, indicating that this population has experienced rapid growth, and those from clade 7 show D >0, indicating that this population has experienced recent bottleneck. *S. invicta* populations from clade 5 presented D > 0 and from clade 6 D < 0, indicating respectively recent bottleneck and rapid growth. In clade 3, as already discussed, populations may be under less human interference and are rarely infected by *Wolbachia* . The populations in clade 6 are probably more affected or influenced by human activity and are also most infected by *Wolbachia* , however clade 3 and 6 are representatives of different species, which may indicate different responses to different selective pressures.

The absence of gene flow may occur due to the existence of a few mtDNA haplotypes that are shared by colonies of different locations. Of the total 72 haplotypes found, only 13 were shared by colonies of different locations. This is evidenced by the size of the circles that indicate the network in Figure 2 (and Table 1 for reference locations). The absence of gene flow may be due to the Paraná River acting as a physical barrier.

The Paraná River is a natural barrier and can restrict gene flow, which could affect the structure and evolution of populations of native fire ants ( Ross et al. 1997 ), but according to Ahrens et al. (2005) there is no explicit information that supports this hypothesis. Likewise, the present study also provides no information to support this hypothesis, although clade 5 included *S. invicta* from northwestern Brazil separated from those of southeastern Brazil. In clade 6, *S. invicta* from Campo Grande (Mato Grosso do Sul) grouped with others from southern Brazil and are therefore separated by the Paraná River, which may mean that mitochondrial gene flow between these regions can occur.

The effects of geographical barriers can be minimized by the constant transport of ants by human activity, resulting in subsequent gene flow. Ahrens et al. (2005) considered that these movements should not be so frequent, as to prevent the continued geographic genetic divergence of populations, but it is important to note that in addition to several species of the genus *Solenopsis* having status as invaders, there is intense trade between certain regions of Brazil, which could explain the occurrence of populations that appear northwest of the Paraná River and in southern Brazil. This may hinder our understanding of the true distribution and evolutionary history of the genus in its region of origin.

This hypothesis finds support in the proposal by Lofgren (1986) and is also emphasized by Tschinkel (2006) , as they reported that human activity was a factor in *S. invicta* dispersal the USA after its introduction. Despite the great diversity of ants in South America, including the *Solenopsis* genus, some species may be favored over others by the reduction of native forests and establishment of monocultures.

Because fire ants have become a global pest, resolving interspecific relationships and species limits is important in understanding the patterns of diversification in South America, their place of origin, and the dispersal of fire ants, especially their distribution in urban or human-influenced habitats. This study shows the need to expand studies using molecular markers in populations of fire ants that occur in urban areas in order to understand the mechanisms these populations are going through and the relationship between rapid urbanization and its relationship with natural populations in these urban areas.

## Abbreviations

COI: cytochrome oxidase I
